# Developing novel evidence-based interventions to promote asthma action plan use: a cross-study synthesis of evidence from randomised controlled trials and qualitative studies

**DOI:** 10.1186/1745-6215-13-216

**Published:** 2012-11-20

**Authors:** Nicola Ring, Ruth Jepson, Hilary Pinnock, Caroline Wilson, Gaylor Hoskins, Sally Wyke, Aziz Sheikh

**Affiliations:** 1 School of Nursing, Midwifery and Health, University of Stirling, Stirling FK9 4LA, UK; 2 Allergy & Respiratory Research Group, Centre for Population Health Sciences, The University of Edinburgh, Edinburgh, EH8 9AG, UK; 3 School of Nursing, Midwifery and Health, University of Stirling, Stirling, FK9 4LA, UK; 4 Nursing, Midwifery and Allied Health Professions Research Unit, University of Stirling, Stirling, FK9 4LA, UK; 5 College of Social Sciences, University of Glasgow, Glasgow, G12 8QF, UK

**Keywords:** Asthma action plans, Cross-study synthesis, Intervention development, Intervention reporting, Qualitative synthesis, Integration

## Abstract

**Background:**

Long-standing randomised controlled trial (RCT) evidence indicates that asthma action plans can improve patient outcomes. Internationally, however, these plans are seldom issued by professionals or used by patients/carers. To understand how the benefits of such plans might be realised clinically, we previously investigated barriers and facilitators to their implementation in a systematic review of relevant RCTs and synthesised qualitative studies exploring professional and patient/carer views. Our final step was to integrate these two separate studies.

**Methods:**

First, a theoretical model of action plan implementation was proposed, derived from our synthesis of 19 qualitative studies, identifying elements which, if incorporated into future interventions, could promote their use. Second, 14 RCTs included in the quantitative synthesis were re-analysed to assess the extent to which these elements were present within their interventions (that is, ‘strong’, ‘weak’ or ‘no’ presence) and with what effect. Matrices charted each element’s presence and strength, facilitating analysis of element presence and action plan implementation.

**Results:**

Four elements (professional education, patient/carer education, (patient/carer and professional) partnership working and communication) were identified in our model as likely to promote asthma plan use. Thirteen interventions reporting increased action plan implementation contained all four elements, with two or more strongly present. One intervention reporting no effect on action plan implementation contained only weakly present elements. Intervention effectiveness was reported using a narrow range of criteria which did not fully reflect the four elements. For example, no study assessed whether jointly developed action plans increased use. Whilst important from the professional and patient/carer perspectives, the integral role of these elements in intervention delivery and their effect on study outcomes was under-acknowledged in these RCTs.

**Conclusions:**

Our novel approach provides an evidence-base for future action plan interventions. Such interventions need to ensure all elements in our implementation model (patient/carer and professional education to support development of effective partnership working and communication) are strongly present within them and a wider range of criteria better reflecting the realities of clinical practice and living with asthma are used to measure their effectiveness. We now intend to test such a complex intervention using a cluster trial design.

## Background

Over recent years, increasing attention has been paid to translating the findings from successful trials into mainstream clinical practice. There is now recognition that if clinical interventions are subsequently to become part of routine healthcare [1] and consistently implemented by different professionals in various settings, ‘real world’ implementation considerations need to be factored in to the design of clinical interventions [1,2].

Worldwide, an estimated 300 million individuals and families are affected by asthma [3]. Asthma action plans are a written or electronic plan for use by patients/carers when their/their child’s peak flow or symptoms change, enabling them to take action in accordance with pre-arranged guidance [4]. These plans, in conjunction with regular review, are effective (for example, they have been shown to reduce unplanned hospital asthma visits [5] and are recommended internationally [3]). Yet, in reality, they are seldom issued by healthcare professionals and, even when issued, remain under-used by patients/carers; the net result being that only a small proportion of those with asthma own or use such plans [6,7]. Given the wide, and long-standing, nature of the gulf between trial-based recommendations and actual day-to-day clinical use, there is an urgent need to identify new, evidence-based approaches to realising the benefits associated with asthma action plan use; and to develop novel interventions predicated on an appreciation of the barriers and facilitators to the issuing of asthma action plans by professionals and their use by patients/carers.

In 2007, we published findings from a systematic review of 14 randomised controlled trials (RCTs) assessing the effectiveness of asthma self-management interventions in promoting use of asthma action plans [8] (see Table 1). Whilst this review provided some good quantitative evidence of interventions effective in facilitating asthma action plan use in trial contexts, for example the Australian 3+ plan which increased the number of asthma action plans issued by professionals [9], there was much less evidence on how best subsequently to initiate and sustain use of these plans by patients/carers once they had been issued [8]. In order to understand better the issuing of these plans by professionals and their use by patients/carers in everyday settings, in 2009 we conducted a qualitative synthesis of 19 studies reporting patient/carer and professional views of self-management interventions containing asthma action plans [10] (see Table 1). (This study also incorporated a linguistic analysis of asthma plan terms and a proposed taxonomy of standardised terms and definitions [4].) From this qualitative synthesis, we highlighted how medically focused asthma action plans were generally not ‘fit for purpose’ because they did not incorporate patient/carer views on asthma or their personal management strategies [10]. We therefore suggested that these plans needed to become more tailored to the needs of individual patients if their use was to be increased and sustained [10].

**Table 1:** Summary of findings from our earlier systematic review of RCTs and qualitative synthesis

1.	*Systematic review of RCT interventions*[8]
	Fourteen studies (15 papers) from six countries:
	- Thirteen trials reported increased action plan promotion (for example, more patients/carers with action plans) and/or use resulting from their intervention. One study reported its intervention had no effect on action plan outcomes [11].
	- Most trials reported interventions which encouraged the promotion of action plans (for example, number of action plans issued by professionals). Few trials measured actual action plan use.
	- Mechanisms encouraging the promotion of action plans included interventions such as the Australian 3+ plan [9] and postal prompts for asthma review with partially completed personalised asthma plans [12].
	- Mechanisms encouraging increased action plan use included the use of a telephone consultation post-hospital discharge by an asthma educator [13].
2.	*Systematic review and qualitative synthesis*[10]
	Nineteen studies (20 papers) from five countries:
	- There is a mismatch between what patients/carers want from action plans and what is currently provided by professionals.
	- The different explanatory models held by patients/carers and professionals towards asthma and its management contribute to this mismatch.
	- To overcome such barriers, asthma plans require to be tailored to patient/carers (for example, customised to their needs and jointly negotiated) and address the wider issues of living with a long-term condition.
	- This requires key elements of communication and partnership working between professionals and patients/carers to encourage shared decision-making, joint negotiation of goals and understanding of their different explanatory models of asthma.

In this present analysis, we sought to integrate the findings from these complementary systematic reviews, to obtain fresh insight into why asthma action plan implementation (that is, the promotion of these plans by professionals and/or their use by patients/carers) was possible within trial settings [8], but was harder to achieve in clinical practice [6,7,14-16]. Of particular importance was enhancing our understanding of how asthma action plans can become more meaningful and useful to those who need to use them on a day-to-day basis. We therefore used the findings from our qualitative synthesis to propose a model of asthma action plan implementation and then ‘tested’ elements in this model by assessing their presence in the interventions evaluated in the RCTs previously identified. Such an approach would, we hypothesised, inform the development of evidence-based interventions that could more easily be incorporated into everyday clinical care settings and the lives of patients/carers.

## Methods

Our cross-study synthesis was guided by the principles of an approach previously used in public health which allows the findings from quantitative and qualitative syntheses to be combined in a systematic review [17,18], using matrices and comparative analysis, and is specifically useful for assessing intervention effectiveness and applicability [17-19]. We adopted a two-staged approach: Stage 1 which consisted of developing a theoretical model of action plan implementation, and Stage 2 where we used the action plan model as a framework for secondary analysis of RCT interventions previously reviewed by us (see Additional File 1).

### Stage 1: Model development

We re-visited our qualitative synthesis, working from our earlier findings [10] to develop a model of asthma action plan implementation (in this context we defined ‘implementation’ as the promotion of these plans by professionals and their use by patients/carers). To do this, we generated a model that incorporated those elements identified in the qualitative synthesis as likely to be important in successfully promoting the use of asthma action plans in practice (see Table 1). In devising our model we had to conceptualise how these elements (for example, communication and partnership-working) would come together in practice, and link to action plan process and outcome measures such as increased use. Model development was informed by team knowledge of the asthma self-management and patient involvement literature as well as multidisciplinary perspectives within the team (general practice, practice and public health nursing). Our model was devised inductively through discussion and the drawing of diagrammatic representations until, after several refinements; consensus was reached on a model of asthma action plan implementation which we thought brought together the key elements of our qualitative synthesis in a way which ‘made sense’ from our perspective as researchers and health professionals and which could then be applied to the RCTs we had previously systematically reviewed.

### Stage 2: Secondary analysis of RCT interventions using our implementation model

Three members of the research team (NR, RJ and CW) critically re-examined the intervention descriptions as reported in the 14 RCTs previously systematically reviewed [9,11-13,20-29] (summarised in Tables 2 and 3) to identify whether they contained the key elements identified in our Stage 1 model and what effect these elements had on study outcome measures. First, for each RCT, general details about the intervention and asthma action plan outcomes were extracted from their reported description onto an Excel database along with specific details relating to the elements within our model. From the extracted data, we determined whether the elements within our model of asthma action plan implementation were present within each of the 14 previously trialled interventions.

**Table 2:** Matrix mapping of elements to the 14 RCT interventions and their outcomes

**Summary of RCT intervention**	**Education for professionals**	**Education for patients/carers**	**Partnership working**	**Communication**	**Number of elements (strong /weak)**	**Effect of intervention on asthma action plan outcomes**
*Intervention resulted in increased asthma action plan (AAP) promotion (for example, more patients with these plans) and use*						
One-off post-hospital discharge telephone consultation by an asthma nurse. Consultation informed by empowerment theory [13]	+	++	+	++	4 (2/2)	↑ promotion & ↑ use
*Intervention resulted in increased AAP use only*						
Structured community centre asthma education promoting behavioural change with follow-up reinforcement at 6 months [20]	+	++	+	++	4 (2/2)	↑ use
Internet-based asthma management tool for patients and physicians included decision support system [21]	+	++	+	++	4 (2/2)	↑ use
*Intervention resulted in increased AAP promotion only*						
Interactive educational seminar for doctors - based on theory of self-regulation - encouraging behavioural change in consultations [22]	++	++	++	++	4 (4/0)	↑ promotion
Proactive, system of care (3+ asthma management plan) included invite for asthma review and education over four practice visits [9]	++	++	+	++	4 (3/1)	↑ promotion
Monthly telephone reinforcement for 1 year by a non-healthcare worker [23]	+	++	++	++	4 (3/1)	↑ promotion
Pre-discharge patient-centred asthma education by a specialist asthma nurse [24]	+	++	++	++	4 (3/1)	↑ promotion
Asthma education (for example, self-management skills) in a community centre by a nurse and non healthcare community workers [25]	+	++	+	++	4 (2/2)	↑ promotion
General practice asthma clinic (included education on asthma management) provided by a nurse and doctor [26]	+	++	+	++	4 (2/2)	↑ promotion
Education (over at least two sessions) by a specialist asthma nurse prior to hospital discharge [27]	+	++	+	++	4 (2/2)	↑ promotion
One-off small group education session encouraging self-management [28]	+	++	+	++	4 (2/2)	↑ promotion
Postal prompts inviting patients for asthma review with intervention groups receiving a partially completed or blank AAP [12]	+	++	+	++	4 (2/2)	↑ promotion
Weekly school-based asthma clinic by a school nurse. Clinic targeted to needs of adolescents [29]	+	++	+	++	4 (2/2)	↑ promotion
*Intervention reported no effect on AAP promotion or use*						
Primary care team quality improvement initiative which included staff coaching and learning [11]	+	+	+	+	4 (0/4)	No effect
**Stage 1 elements: Strong presence**	2	13	3	13		
**Weak presence**	12	1	11	1		
**No presence**	-	-	-	-		
**Total**	14	14	14	14		

+, Weak presence; ++, Strong presence; 0, No presence.

**Table 3:** The 14 RCT interventions with their elements and detailed study outcomes

**Intervention**	**Elements strong/weak**	**RCT quality assessment**^**a**^	**Summary of trial interventions asthma action plan (AAP) outcome measures**
	**Increased AAP use at 6 to 12 months post-intervention**		
Structured community centre asthma education promoting behavioural change with follow-up reinforcement at 6 months [20]	2/2	C	Increased AP use @ 1 year:
			- Significantly higher AAP use (*P*=0.008) than the control group. And, approximately 68% reported willingness to adjust medications.
One-off post-hospital discharge telephone consultation by an asthma nurse. Consultation informed by empowerment theory [13]	2/2	C	Increased and promoted AAP use @ 6 months:
			- More participants with AAP than control group: 88% *vs.* 72% (*P*=0.001)
			- Greater frequency of AAP use than in control group: 32% used often versus 22% & 56% used occasionally (*vs.* 51%)
Internet-based asthma management tool for patients and physicians with decision support system [21]	2/2	C	Increased AAP use @ 6 months:
			- More participants used an Internet-based AAP (88%) than an AAP from a specialist (66%) or from a GP (6%) (*P* <0.001)
	**Promoted AAP use at 1 to 2 years post-intervention**		
**Interactive educational seminar for doctors aimed at encouraging behavioural change during their clinical consultations**[22]	**4/0**	**C**	Promoted AAP use @ 2 years:
			- More parents had written doctor information about changing medicines in response to symptom changes (*P*=0.05)
			- Doctors commended parents for taking right asthma management actions (*P*=0.02), enquired about parent medication fears/concerns (*P*=0.02), explained the short-term therapeutic plan (*P*=0.03) and made it easier for families to follow medication instructions (*P*<0.004)
**Proactive, system of asthma care (3+ asthma management plan) including invites for asthma review and patient education**[9]	**3/1**	**A**	Promoted use @1 year:
			- More children had an AAP (44% *vs.* 34%; OR 2.2 95% CI 1.2-4.1)
**Pre-discharge asthma education by a specialist asthma nurse**[24]	**3/1**	**B**	Promoted use up to 1 year:
			- At 1 month: More patients had an AAP (*P* <0.0001)
			- On re-admission to hospital up to 1 year: more patients had an AAP (*P* <0.0001)
**Monthly telephone reinforcement for 1 year by a non-healthcare worker**[23]	**3/1**	**C**	Promoted use at @ 1 year:
			- More than 70% of participants reported improved understanding of AAP use
Postal prompts inviting patients for asthma review with intervention groups receiving a partially completed or blank AAP [12]	2/2	B	Promoted use @1 year:
			- More participants reported increased patient understanding of how to use AAP (OR 2.20, 95% CI 1.13-4.30) and usefulness of their AAP (OR 2.65, 95% CI 0.87-7.99)
	**Promoted AAP use at less than 1 year post-intervention**		
One-off small group education session encouraging self-management [28]	2/2	C	Promoted AAP use @ 10 months:
			- AAP ownership higher (*P* <0.001)
Asthma education (for example, self-management skills) in a community asthma education centre by a nurse and non-healthcare community workers [25]	2/2	C	Promoted AAP use @ 9 months:
			- More children (*P*=0.0001) and adults (*P*=0.01) with AAP
			- Better knowledge of action in response to gradually worsening asthma (for adults *P*=0.005 and parents *P* >0.05) and suddenly worsening asthma (adults *P* <0.01)
General practice asthma clinic (including asthma management education) provided by nurse and doctor [26]	2/2	C	Promoted AAP use @ 6 months:
			- More in intervention group (75%) had written AAP (*vs.* 65% of controls). When adjusted for baseline measures and clustered by doctor, OR of 1.62 (95% CI 0.82-3.22)
Education (over at least two sessions) by a specialist asthma nurse prior to hospital discharge [27]	2/2	C	Promoted AAP use @ 6 months:
			- 86% of intervention group had AAP *vs.* 17% of control group (*P* <0.01). Greater numbers ‘chose self-management’, for example, increased inhalers in intervention group compared to controls (77% *vs.* 57% *P* <0.01)
Weekly school-based asthma clinic by a school nurse. Clinic targeted to needs of adolescents [29]	2/2	C	Promoted AAP use @ 6 months:
			- More in intervention group had an AAP (*P* <0.001; OR varied between schools (*P*=0.01)
	**Had no effect on AAP promotion or use at 1 year post-intervention**		
Primary care team quality improvement initiative which included staff coaching and learning [11]	0/4	C	No effect on AAP promotion or use @ 1 year

The four interventions in **bold** text indicate those assessed as containing three or more strongly present elements.

^a^The quality assessment process is also reported elsewhere [8]. Briefly, Quality Grade A= low risk of performance, attrition and detection bias. Grade C= high risk of bias.

Second, having identified which element(s) were contained within each intervention, we determined their strength of presence. Employing a recognised methodology [2], we adopted a qualitative consensus rating approach using a three-level rating scale, adapting the scale so that an element’s strength of presence could be rated as: ‘strong’, ‘weak’ or ‘no’ presence. Decisions about an element’s strength of presence were based on our interpretation of the original authors’ trial descriptions and reporting. To promote consistency in this subjective rating process we developed inter-rater guidance (Table 4). This guidance was refined as the rating process progressed with additional points of clarification added as necessary. The ratings process was therefore iterative as any refinements to the rating guidance required a review of earlier decisions to ensure they were congruent. This assessment was completed by the three researchers working independently and then in pairs to compare findings and check accuracy of extracted data. For each RCT intervention, these decisions were recorded onto the Excel database. Any disagreements in rating were discussed within the rating subgroup until agreement was reached. If there was still disagreement, issues could be referred to the wider team for resolution, but in the event this did not prove necessary.

**Table 4:** Guidance for assessing element presence within the 14 RCT interventions

*Education for professionals:*	A wide range of activities were assessed as containing this element. For example, this element was considered to have a:
	- Weak presence: if practitioners were simply instructed in delivering the intervention or education delivered was restricted to those providing the intervention but they were not ‘mainstream’ practitioners.
	- Strong presence: if the education delivered was more complex and promoted behavioural change (for example, practitioners were taught to change their consultation style and/or there was subsequent reinforcement of professional education and/or education was offered to mainstream practitioners, not just those delivering the intervention).
*Education for patients/carers:*	A wide range of activities were assessed as containing this element. For example, this element was considered to have a:
	- Weak presence: if patients/carers were simply offered/given asthma education as a one-off event and/or there was no/little evidence that this education was tailored to the needs of individual patients/carers.
	- Strong presence: if patients/carers received in-depth asthma education tailored to their need. Such education could be delivered as a one-off session or on more than one occasion.
*Partnership working:*	For example, this element was considered as having a:
	- Weak presence: if an intervention simply increased the opportunities for professionals, patients and carers to come together to discuss asthma and its management.
	- Strong presence: If in the increased opportunities for asthma review, professionals actively encouraged patients/carers to participate in their asthma reviews and/or action plans were jointly developed. Interventions also encouraged patient/carer empowerment/enablement and opportunities for patient/professional partnership working were encouraged longer term.
*Communication:*	For example, this element was considered as having a:
	- Weak presence: if an intervention simply provided patients/carers with additional opportunities to discuss their/child’s asthma with a professional (for example, a new asthma clinic was established). Strong presence: if the intervention improved the quality of asthma communication between patients/carers and professionals (for example, professionals actively listened to patients, encouraged patients to express their fears/anxieties and responded to these).

Third, once each RCT intervention had been examined to determine which elements it contained and their strength had been assessed using the three-level rating scale (that is, strong, weak or no presence) the findings were mapped onto a matrix. Matrix mapping has previously been used to integrate controlled trials and qualitative studies in systematic reviews [17,18,30] as it enables these different sets of findings to be ‘juxtaposed’ [18]. Our synthesis matrices mapped which elements within our qualitatively derived model were components within these 14 RCTs interventions and in what strength, linked to each trial’s quality assessment and asthma action plan outcomes (Tables 2 and 3). Matrix mapping enabled us to illustrate visually the strength to which the elements within our model were present in the 14 trialled interventions (Table 2) and facilitate comparative analysis of element presence on intervention asthma action plan outcomes (Table 3), enabling a fresh interpretation of these 14 RCT interventions and their delivery to emerge. Findings are presented in these matrices and a descriptive narrative.

## Results

### Stage 1: Model development

Our proposed model (see Additional File 2), generated from the qualitative synthesis of patients/carers’ and professionals’ views, contained four elements likely to be essential in supporting asthma action plan implementation (in bold below and Table 5). In summary, asthma action plan implementation was considered to require: **professional education and patient/carer education** to encourage more **effective communication and partnership** working during asthma consultations. Such an approach would enable professionals and patients/carers to understand better each other’s views on asthma, and its management, helping to facilitate shared decision-making including joint development (and/or review) of action plans more suited to the needs of patients/carers. We hypothesised that as more such asthma action plans are issued, parents/carers would perceive these as more meaningful and relevant to their needs, their use would increase as a consequence, encouraging professionals to issue these more often.

**Table 5:** Details of the elements within the asthma action plan implementation model

*Education for health professionals, for example:*	
·	Initiatives to facilitate change in their asthma attitudes, beliefs and/or behaviours such as professionals actively encouraging patient/carer involvement in asthma decision-making and/or valuing the personal experience of those living with asthma.
·	Education encouraging professionals to customise asthma action plans with patients/carers and enabling professionals to understand that professionals and patients/carers may have different models of asthma and its management.
*Education for patients/carers, for example:*	
·	Education targeted to the patient’s/carer’s stage in the ‘learning to manage’ process. Includes instruction on medications, recognition of symptoms, avoiding triggers within the context of asthma self-management and the more holistic issues of living with asthma.
·	Education to encourage patients/carers to actively participate in the joint development/review of their asthma action plans and enabling them to participate in shared decision-making.
*Encouraging partnership working, for example:*	
·	Continuity of asthma care to provide on-going opportunities for asthma education and promoting patient/carer empowerment.
·	Professionals encouraging good working relationships with patients/carers (for example, by promoting active involvement and shared decision-making within asthma consultations such joint development of action plans).
·	Initiatives to encourage patients/carers and professionals to understand different models of asthma management.
*Effective communication, for example:*	
·	Professionals actively seeking and listening to patients/carers asthma experiences, their management strategies and asthma anxieties. Professionals offering additional opportunities for patient asthma review.
·	Professionals responding to the above, for example, through targeting of patient/carers asthma education, encouraging joint development of tailored asthma action plans.

### Stage 2: Secondary analysis of RCT interventions using our implementation model

Table 2 summarises the 14 interventions [9,11-13,20-29] and broadly categorises each intervention’s effect on asthma action plan implementation (that is, increasing promotion and/or use). This table also illustrates which interventions contained the four identified elements and to what strength (that is, ‘strong’, ‘weak’ or ‘no’ presence).

### Presence of essential elements in trial interventions

The four elements likely to support asthma action plan implementation were present in all 14 RCT interventions, but to varying degrees (Table 2). Although some components of the complex interventions (such as asthma clinics and systems of asthma review) were well documented this was not usually the case with the elements, even in those trials we previously assessed as being of high quality [8]. Frequently, where key information about the elements was provided this was scant and ambiguous. For example, interventions stating that patients were ‘encouraged to actively participate’ [27] did not clarify what this actually meant regarding communication and joint development of asthma action plans. So although it was relatively straightforward to determine whether an element was contained in each intervention, it was harder to assess its strength of presence (Table 2). Only one intervention [22] had all elements assessed as strongly present and in terms of providing a detailed intervention description, this was an exemplar paper.

Overall, the elements of patient/carer education and communication were easiest to identify from the trial descriptions and were assessed as strongly present in nearly all interventions (Table 2). By comparison, partnership working and professional education were less well described and were generally considered weakly present (Table 2). In the case of professional education there were several reasons for this finding. Trial interventions frequently detailed patient/carer education, but did not specify what education, if any, was also given to professionals. So, if an intervention was delivered in a general practice, patient education would be well described, but not how practice staff were also taught to support this initiative. Alternatively, professional education was limited to those delivering an intervention, but excluded other co-workers in a study. One such example used trained community workers to deliver asthma education to children and parents, but doctors who also saw these families received ‘no special instruction’ [25]. On other occasions, professional education was clearly a component of an intervention as trial descriptions noted professionals were ‘given information’ [20] but, again, difficulty in interpreting the meaning of terms such as ‘training’, may have resulted in an element’s strength of presence being under- or over-rated. Consequently, only two interventions [9,22] were assessed as containing this element to a strong extent - in both cases professional education was considered to extend beyond simple instruction to encourage sustained changes in professional behaviour [9,22]. Importantly, these two interventions (an interactive medical seminar [22] and the proactive 3+ plan of asthma care [9]) promoted asthma action plan implementation by changing the behaviour of mainstream clinical practitioners unlike some other interventions where professionals delivering the intervention were often affiliated to the research study.

### Intervention effectiveness and the presence and strength of essential elements

Thirteen interventions reported increasing asthma plan implementation by, for example, increasing the number of these plans issued and/or used. As each of these interventions contained all four elements (Table 2), there was an association between intervention effectiveness and element presence. However, whilst all elements needed to be present in an intervention to support its implementation, at least two of these needed to be strongly present for the intervention to be effective (Table 2). This finding is also supported by the only RCT reporting its intervention (a primary care-based quality improvement initiative) had no overall effect on asthma plan promotion and/or use [11] as this was the sole intervention with all elements assessed as weakly present. As four studies providing data for the longest period post-intervention (one to two years) [9,22-24], contained three or more strongly present elements, it also appears that strength of element presence helps to sustain behavioural change and support asthma action plan implementation longer-term, for example, through more doctors commending parents/patients for taking the right asthma management action(s) [22].

Interestingly, most trials (*n*=9) reporting intervention effectiveness did so using a narrow range of criteria relating to the promotion of asthma action plans such as the number of patients/carers issued with plans rather than numbers using them. Only four studies [12,22,23,25] reported measures that related specifically to the elements, for example whether education resulted in more patients/carers understanding asthma action plan use post-intervention (Table 3). No study reported whether patients/carers worked with health professionals to develop or review their asthma action plans and, if so, whether this partnership resulted in patients/carers considering these plans to be more relevant or meaningful to their needs, thereby increasing their use. Variations in reported study process and outcome measures also meant it was not possible to specify what effect the individual elements had on asthma action plan implementation, that is, whether an intervention with three strongly present elements had better asthma action plan outcomes than an intervention with only two strong ones.

## Discussion

We generated a model of asthma action plan implementation derived from the views of patients/carers and professionals. This model suggested that elements such as partnership working are ‘active ingredients’ [31] contributing to an intervention’s effectiveness in trial settings. We then used this model as a framework to re-analyse 14 previously conducted RCTs and found that whilst these four elements were present within these interventions, they were not always explicitly described in their intervention descriptions and their contribution to an intervention’s delivery was not fully acknowledged and/or their effect on asthma action plan implementation measured.

### Strengths and limitations

Synthesising quantitative and qualitative research is still relatively uncommon and our analysis is an early example of its use in understanding the management of long-term conditions, specifically asthma [32]. Bringing diverse sources of evidence together so they can be examined [33] is an emerging but complex field [32] because several approaches exist including thematic synthesis [34] and critical interpretive synthesis [35]. However, our approach is novel for two reasons. First, the level of interpretation and analysis in our cross-study synthesis went beyond ‘summarising’ existing evidence [36] and reporting of themes arising from the original studies. Instead qualitative studies were synthesised and ‘subsumed’ into a ‘higher order theoretical structure’ [37], through generation of our action plan model. As such, we conducted third level analysis, providing a new interpretation of the original authors’ findings [38]. This differentiates our approach from others which simply aggregate findings from individual studies, such as integrative reviews [39-41].

Second, our work differs from the public health studies which guided us because these synthesised the qualitative research, producing themes or recommendations which were then integrated with trials [17,18] to ‘draw out implications’ [30] such as which trial interventions ‘matched’ the qualitative themes and with what effect [18]. By comparison, the elements in our qualitatively derived model of action plan implementation were ‘tested’ against intervention components to better understand their delivery. In the public health studies [17,18] trial and qualitative evidence are synthesised and integrated concurrently, whereas we conducted our quantitative and qualitative syntheses separately before combining them. In this innovative way our qualitative synthesis provided an alternative framework with which to secondary analyse previously reviewed RCT interventions. This new analytical perspective derived from the ‘real world’ experiences of asthma action plans by patients/carers and professionals enabled a more nuanced appreciation to emerge of why asthma action plan implementation was more achievable in these RCTs than it in clinical settings, an insight which was not previously possible based on quantitative evidence alone.

Within the time and resources available, it was not possible to externally validate our model, but this is a useful area for future research. The elements in our model were also considered as separate entities, whereas in practice they are inter-related, and were applied retrospectively to previously trialled interventions. Inadequately detailed or ambiguous intervention descriptions relating to these elements were anticipated at the outset; however, these were greater than expected given the central importance of communication and collaboration during asthma consultations [42,43]. Whilst intervention descriptions were often superficial, it was not feasible within our study timescales to contact original authors for more details. In terms of resources, our cross-study synthesis was similar to conducting a systematic review that is, it took part-time researchers about 6 months to devise our model, extract data, construct matrices and interpret findings. This might seem resource intensive given we were integrating qualitative and quantitative findings from two previously conducted systematic reviews however, this mixed method integrative approach enabled us to ‘fully exploit the potential’ of our earlier work [44], allowing us to ‘learn more’ from our previous data [45].

### Implications for asthma action plan interventions

For those developing complex interventions, their intervention choices should be based on the relevant theory [46] and understanding of underlying causal processes and mechanisms [47]. We now have the theoretical basis for a complex intervention to promote asthma action plan use in primary care and intend to test it within a cluster trial design. However, if we had proceeded to trial stage earlier, based solely on findings from our trial-based systematic review, without first utilising existing qualitative studies, we would have developed an intervention which did not adequately take into account the views and needs of those who should be issuing and/or using these plans. Consequently our planned intervention would not have adequately reflected all four elements or equally emphasised the organisational and interpersonal contexts in which asthma care is delivered. The effectiveness of our intervention would also have been evaluated by measurement against a narrow range of criteria which did not adequately reflect the patient/carer and professional experience of asthma action plan implementation in practice. Our approach thus illustrates how the knowledge gained from qualitative and quantitative studies can be brought together through cross-study synthesis to help provide the necessary theoretical foundations for the development and evaluation of future complex interventions [31,48] ensuring future interventions are ‘evidence-based rather than evidence-inspired’ [47].

Our findings from this and our earlier studies [8,10] suggest that future interventions to encourage asthma action plan implementation need to reflect our model. In particular, ensuring they embed the joint development of these plans by professionals and patients/carers within routine systems of regular asthma review delivered by mainstream practitioners. In addition, education targeted towards professionals and patents/carers is needed to facilitate behavioural change by supporting development of the necessary communicative and collaborative skills, such as negotiation and goal setting. For pragmatic reasons, the asthma review system would need to reflect local and national review processes (such as the UK Quality and Outcomes Framework [49]).

### Implications for self-management interventions

By making visible the full nature of the ‘collective action’ that is, the ‘work’ professionals did to make these trial interventions ‘function’ [1], especially their interpersonal work with patients/carers, our study has relevance to the pragmatic development, reporting and evaluation of self-management interventions generally. In particular, reinforcing the importance of the individual clinical context in complex interventions [50], highlighting the need for it to be explicitly acknowledged.

In our analysis, from the patient/carer perspective a professional’s skills in, for example, partnership working were important in facilitating intervention implementation yet, such features were generally ‘unseen content’ [51] of these 14 interventions, rather than visible integral components. The issue of limited intervention description [52] and the need for a ‘detailed account of what was done’ [53] including ‘critical techniques’ [47] employed during an intervention is recognised [52]. Whilst guidance on the quality, consistency and transparency of intervention descriptions is available [51,52,54-57], our investigation highlighted that in addition to details of the ‘who, what, when and where’ of an intervention [52], researchers also need to detail ‘in what way’ an intervention was delivered, specifically how professionals actually engaged with patients/carers in their clinical consultations. Critically, the meaning of such intervention terms needs to be clearly defined.

Uncertainty of meaning of terms such as ‘counselling’ [56] and ‘tailoring’ [52] are already recognised, but we found a much wider range of terms lacked clarity, especially terms relating to the ‘active’ participation of patients/carers. Our study adds further support to the call for a ‘common language in intervention description’ [51] but also identifies other areas where clarification is required in terms of the contextual factors which could impact on the delivery of an intervention. In particular, researchers need to specify what interpersonal skills and attributes those professionals delivering their interventions bring with them into the research setting as their ‘usual’ [56] pre-existing skills in communication and collaboration, determine the clinical contexts they create, in turn influencing intervention implementation. Where future interventions are delivered by a ‘typical’ healthcare provider [58] or a practitioner with ‘extensive expertise’ [57], researchers should also specify what this means in terms of interpersonal clinical skills. For example, researchers could rate on a scale from 0 to 100 [2,59] or on a ‘continuum of weak to strong’ [60] the extent to which professionals delivering their intervention were, for example, already sharing decision-making with patients or empowering them to self-manage pre-intervention. We appreciate that clarifying such terms calls for even more detailed descriptions of complex interventions, but such additional information could, as others have suggested, be provided in online supplementary information [54,56].

## Conclusions

The development of theoretically robust complex healthcare interventions which better reflect the experience of those using those interventions in ‘real life’ are required. Using findings from a qualitative synthesis of patients/carers and professionals views to generate a model of asthma action plan implementation, then testing this against 14 interventions enabled us to obtain fresh insight into how these trials encouraged asthma action plan intervention implementation in research settings. In particular, we identified the importance of four elements (professional education, patient/carer education, professional/patient/carers partnership working and communication) likely to be essential in facilitating the ‘right’ individual contexts between patients/carers and professionals to support implementation. Given that the evidence for these elements was derived from the views of those who should be issuing and/or using asthma action plans, we now have a clearer understanding of why there has been a longstanding gap between recommended and actual asthma plan use and why the promotion and use of asthma plans has been easier to achieve in research rather than clinical settings. That is, whilst the presence and strength of presence of the four elements contributed to asthma action plan implementation in trial settings, these elements may have been absent from, or inconsistently available, in everyday practice settings, thereby reducing the potential for clinical implementation.

Our asthma action plan specific study has relevance to self-management interventions more generally. First, there is a need for future such interventions to include partnership working and communication supported by professional and patient/carer education. These four elements need to be explicitly and unambiguously detailed in the description of self-management interventions. In turn this will enable the clear identification of those features, such as a professional’s consultation style, which can enhance the individual contexts of a research setting, facilitating implementation. Second, we identified an alternative means in which qualitative evidence can contribute to intervention development and evaluation by, for example, identifying study process and outcome measures reflecting the perspective of patients/carers and professionals, not just the researcher perspective. Finally, we believe our novel approach is a rigorous and systematic means of identifying the underpinning theory, components and outcome measures necessary to support the design and evaluation of future evidence-based complex interventions [31,48].

## Competing interests

HP chairs the Patient education and self-management Evidence Review Group of the British Thoracic Society/Scottish Intercollegiate Guideline Network asthma guideline. AS is a past chair of the British Thoracic Society’s Science and Research Committee and a member of its Council and Executive. No other authors report competing interests.

## Authors’ contributions

All participated in designing the study, the grant funding application, project steering group meetings and electronic discussions throughout the study and in drafting this paper. NR, RJ, CW, HP and AS developed the Stage 1 theoretical model. Stage 2 processes (for example, extracting data, checking for accuracy, essential element rating, matrix mapping and comparative analysis) were conducted by NR, RJ and CW with SW providing supervision. All authors contributed to the interpreting of findings and conceptual analysis required during the drafting of this paper. All authors read and approved the final manuscript.

## Supplementary Material

Additional file 1 The overall process of cross-study synthesis. Click here for file

Additional file 2 Asthma action plan implementation model derived from qualitative synthesis. Click here for file
