# Supplementary material for: Developing novel evidence-based interventions to promote asthma action plan use: a cross-study synthesis of evidence from randomised controlled trials and qualitative studies
Source: Trials. 2012 Nov 20;13:216. doi: 10.1186/1745-6215-13-216 (PMC3561124; doi:10.1186/1745-6215-13-216)
Supplement: Additional file 1 — The overall process of cross-study synthesis. [file 1745-6215-13-216-S1.doc]

**Diagram 1: The overall process of cross-study synthesis***

*Diagram adapted from [14;15]

**Research Questions**

What are the barriers and facilitators to asthma action plan promotion and/or use?

Can a model of asthma action plan implementation be identified and, if so, what are its elements?

What would be the components of a future evidence-based primary care action plan intervention?

**Synthesis 1:**

**Systematic Review of Randomised Controlled Trials (RCTs) [8]**

Application of inclusion criteria

Quality assessment

Data extraction

Quantitative narrative synthesis (14 studies/15 papers)

Interventions reporting increased action plan promotion and/or use (13 studies)

**Synthesis 2:**

**Synthesis of Qualitative Studies reporting patient/carer and professional views [10]**

Application of inclusion criteria

Quality assessment

Data extraction

Thematic analysis of participant views (19 studies/20 papers)

Synthesis of conceptually rich papers (8 studies)

**Synthesis 3:**

**Integrating findings from the quantitative and qualitative syntheses**

*Stage 1:* Development of a model of asthma action plan implementation - including its key elements – derived from the findings from Synthesis 2.

*Stage 2:* Matrix mapping and comparative analysis to determine whether the elements contained in the model of asthma action plan implementation were found in the Synthesis 1 RCT interventions and what effect these elements had on trial outcomes.

*Diagram adapted from (14;15)
