# Supplementary material for: Developing novel evidence-based interventions to promote asthma action plan use: a cross-study synthesis of evidence from randomised controlled trials and qualitative studies
Source: Trials. 2012 Nov 20;13:216. doi: 10.1186/1745-6215-13-216 (PMC3561124; doi:10.1186/1745-6215-13-216)
Supplement: Additional file 2 — Asthma action plan implementation model derived from qualitative synthesis. [file 1745-6215-13-216-S2.doc]

**Diagram 2: Asthma action plan implementation** model derived from qualitative synthesis findings

| Intervention implementation stage | The four elements likely to be essential in promoting asthma action plan implementation are shown in grey below and outlined in Box 3. |
| --- | --- |
| *Pre- intervention delivery*  Education for Health Professionals (HP) e.g. on shared decision-making, joint goal setting  (*If required – depends on pre-existing clinical conditions)* | The availability of pre-existing factors* which could enhance the local contexts of asthma action plan (AAP) implementation    Education for Patients/Carers (P/C) e.g. shared decision-making, joint goal setting |
| *Delivery*  *of*  *intervention*  AAP jointly developed and/or reviewed by P/C and HP.  AAP more meaningful/relevant to the needs of P/C e.g. includes their personal asthma management strategies | Communication  (Effective and two-way between HP & P/C)  Partnership working between HP & P/C e.g. sharing decision-making and goal setting |
| *Post-intervention*  🡩 AAP issued by HP to P/C  🡩 HP working with P/C to develop/review AAP  🡩 HP seeing AAP as relevant and useful  *delivery*  *measures*  *e.g.* | 🡩 AAP used by P/C  🡩 AAP reported by P/C as meaningful, fit for purpose & suited to their needs  🡩 P/C participating in AAP development and review |
| Notes: *Pre-existing factors which could enhance local contexts of implementation include professionals with advanced skills in promoting self-management e.g. empowering patients, and/or sharing decision-making or clinical areas e.g. training general practices that may be more receptive to change than other settings. | |
